# Integrated web visualizations for protein-protein interaction databases

**DOI:** 10.1186/s12859-015-0615-z

**Published:** 2015-06-16

**Authors:** Fleur Jeanquartier, Claire Jean-Quartier, Andreas Holzinger

**Affiliations:** Research Unit HCI-KDD, Institute for Medical Informatics, Statistics and Documentation, Medical University Graz, Auenbruggerplatz 2/V, Graz, 8036 Austria; Institute for Information Systems & Computer Media Graz University of Technology, Inffeldgasse 16c, Graz, 8010 Austria

**Keywords:** Visualization, Visual analysis, Network visualization, Protein-protein interaction, Systems biology

## Abstract

**Background:**

Understanding living systems is crucial for curing diseases. To achieve this task we have to understand biological networks based on protein-protein interactions. Bioinformatics has come up with a great amount of databases and tools that support analysts in exploring protein-protein interactions on an integrated level for knowledge discovery. They provide predictions and correlations, indicate possibilities for future experimental research and fill the gaps to complete the picture of biochemical processes. There are numerous and huge databases of protein-protein interactions used to gain insights into answering some of the many questions of systems biology. Many computational resources integrate interaction data with additional information on molecular background. However, the vast number of diverse Bioinformatics resources poses an obstacle to the goal of understanding. We present a survey of databases that enable the visual analysis of protein networks.

**Results:**

We selected M =10 out of N =53 resources supporting visualization, and we tested against the following set of criteria: interoperability, data integration, quantity of possible interactions, data visualization quality and data coverage. The study reveals differences in usability, visualization features and quality as well as the quantity of interactions. StringDB is the recommended first choice. CPDB presents a comprehensive dataset and IntAct lets the user change the network layout. A comprehensive comparison table is available via web. The supplementary table can be accessed on http://tinyurl.com/PPI-DB-Comparison-2015.

**Conclusions:**

Only some web resources featuring graph visualization can be successfully applied to interactive visual analysis of protein-protein interaction. Study results underline the necessity for further enhancements of visualization integration in biochemical analysis tools. Identified challenges are data comprehensiveness, confidence, interactive feature and visualization maturing.

## Introduction and Motivation

Both, wet and dry scientists in the domains of Bioinformatics and Life Sciences have to deal with huge amounts of data on protein-protein interactions (PPIs) to understand human life. They have to rely on comprehensive data from web resources. Getting an overview is crucial. Visualization supports this complex task. There are numerous web resources and databases. But assessments of individual strengths and weaknesses of the available resources are scarce. In this paper, we evaluate identified resources in regard to the support of integrated visualization and highlight promising examples. To our knowledge there is no such up-to-date comparative study.

Proteins are the building blocks of life. Interactions between proteins determine cellular communication. Signal transduction cascades process information of various stimuli for a cell to respond to external signals. Cell signaling is based on molecular circuits consisting of receptor proteins, kinases, primary and secondary messengers. Together, they modulate gene transcription or the activity of other proteins [1].

Studies on these complex interaction networks give insight into life-determining processes and can be used for combating disease. Therefore, large datasets are used that contain information on PPIs gained from experiments using yeast two-hybrid systems as well as affinity-bait systems [2]. Computational tools for uncovering PPIs are based on the comparison of large-scale experiments, literature curation, text-mining and computational prediction results of protein interactions. These tools are available to the public via online databases [3]. There are numerous software tools and huge databases of PPIs used to gain new insights into systems biology. While many Bioinformatics resources integrate interaction data with other types of information, visualization plays a major role in the process of understanding and sense-making [4–6].

In the last decade, experts started to integrate possibilities for visualization of PPI networks to facilitate exploration and analysis tasks. Visualizations of interaction networks are mostly rendered graphs providing an overall picture of pathways mapping biological functions [7–10].

Some of the many available resources lack maintenance and input of updates. Most of all, they lack usability [4, 5, 11]. The question remains: Which tool is the best choice for the analysis task at hand? Many analysts in the field of Biochemistry manually mine text. They try to find information on related studies and search for appropriate tools. Many researchers do not know which resources are available and which one is best suited to support their analysis. From a computer science perspective there are many possibilities to facilitate the analysis process, particularly making use of visualization features to fully exploit the human capabilities of information processing and pattern perception [12]. To support analysts in Biochemistry it is crucial to pick the right tool for the task at hand [6, 11]. We, therefore, highlight a small set of tools, available on the web, that integrate auxiliary visualization features. The study focuses on web page integrated visualization software that uses the most common technologies supported by current standard web browsers. Online solutions offer fast and easy utilization characteristics compared to client standalone tools. By making use of web visualization tools we overcome issues with standalone solutions including the complicated task of finding and installing third-party solutions, appropriate plugins, difficulties in retrieving biological data, finding appropriate information when searching in default databases that are too generic within local standalone solutions, lack of central storage, interchange and collaboration possibilities [10, 13]. Web visualization represents a field of research on its own finding solutions for limitations in speed, interoperability and navigation. Hence, interdisciplinary scientists improve Bioinformatics databases and tools by adding biological content as well as integrating pervasive web applications featuring graph-based information representation. Interaction and export options are integrated into online tools for further processing of graphs with standalone tools including Cytoscape or Navigator for high computing analysis tasks [9, 10, 14–16]. Standalone tools offer the possibility of individual upgrades in form of add-ons and plugins, numerously available online. Changes to web tools have to be implemented by the provider. Computing power and capacity constitute limiting factors for both web and standalone products. Cytoscape represents a software, most commonly used by bioinformaticians. Still, covering this topic goes beyond the scope of this work. We focus on software that can be easily accessed and used by all experimentalists who deal with PPI analysis. We focus on web software, that neither requires any particular system, nor any root rights, any user’s knowledge of system administration or how to install a particular software.

We start with giving some background on visualization in PPI analysis. Then present the comparison study and summarize comparison results of identified tools that suite the task of interactive visual analysis. At last we present its’ discussion and identified challenges.

## Background

The human genome contains over 20000 protein-coding genes, while the total number of different proteins is still unknown and estimated to be much higher [17, 18]. Comprehensive knowledge of protein interactions represents the key to understanding the underlying functional network. The molecular organization can be visualized as a network of differentially connected nodes. Each node stands for a protein and edges represent dynamic interactions. Nodes thereby receive input and output values as mathematical functions [19].

Computational results can be analyzed by interactive visualizations. The integrated process of Visual Analytics is essential to sensemaking in Life Sciences. Analyzing a problem in a visual way allows to highlight certain features that are not perceptible otherwise [4, 5, 11, 12].

There are several tools for PPI visualization that not only deal with the general questions of PPI analysis but focus on structural analysis of particular protein domains and peptide sequences (e.g. PDB that archives a large amount of macromolecular structural data that can be visualized). Furthermore, many resources are domain specific and do not support the analysis of the entire interactome (e.g. “NIA”, a Mouse PPI Database, or PFAM, a collection of protein domains). The interactome incorporates proteins as well as other chemical compounds as ions, nucleic acids, in sum all interacting elements. In this work, we focus on general resources for PPI analysis that integrate tools for visualizing parts of the human proteinogenic interactome as PPI network.

Graph drawing represents the traditional way of visualizing interactions. Graph visualizations constitute a well-known, sophisticated method in computer science [14]. There are many different well-established and evaluated layout algorithms for node arrangement in graphs. Force-directed layouts are the main algorithms used for graph drawing. As a result related nodes are placed closer to each other, and highly connected protein interactors as well as clusters of interactors are easily identifiable. Current network visualization resources make use of visualization libraries. One example is the Flash version of Cytoscape [20], that is used n the tool IntAct [21] among others. Additionally, JavaScript (JS) based visualization libraries are currently emerging, including BioJs [22], that is used in PINV [23]. Cytoscape.js is a successor of Cytoscape Web and there is also a wrapper for using cytoscape in BioJs [22].

However, there are several issues and open problems when visualizing biological networks [24, 25]. Nodes are connected through edges representing underlying interactions and should provide interactivity for supporting exploration [26]. Standalone tools like Gephi, Navigator or Cytoscape include various modifications and settings for such purposes. In case of (web-based) graph rendering there are several challenges regarding the handling of large graphs, when dealing with high levels of details and interaction features [16, 26, 27].

**Fig. 1:**
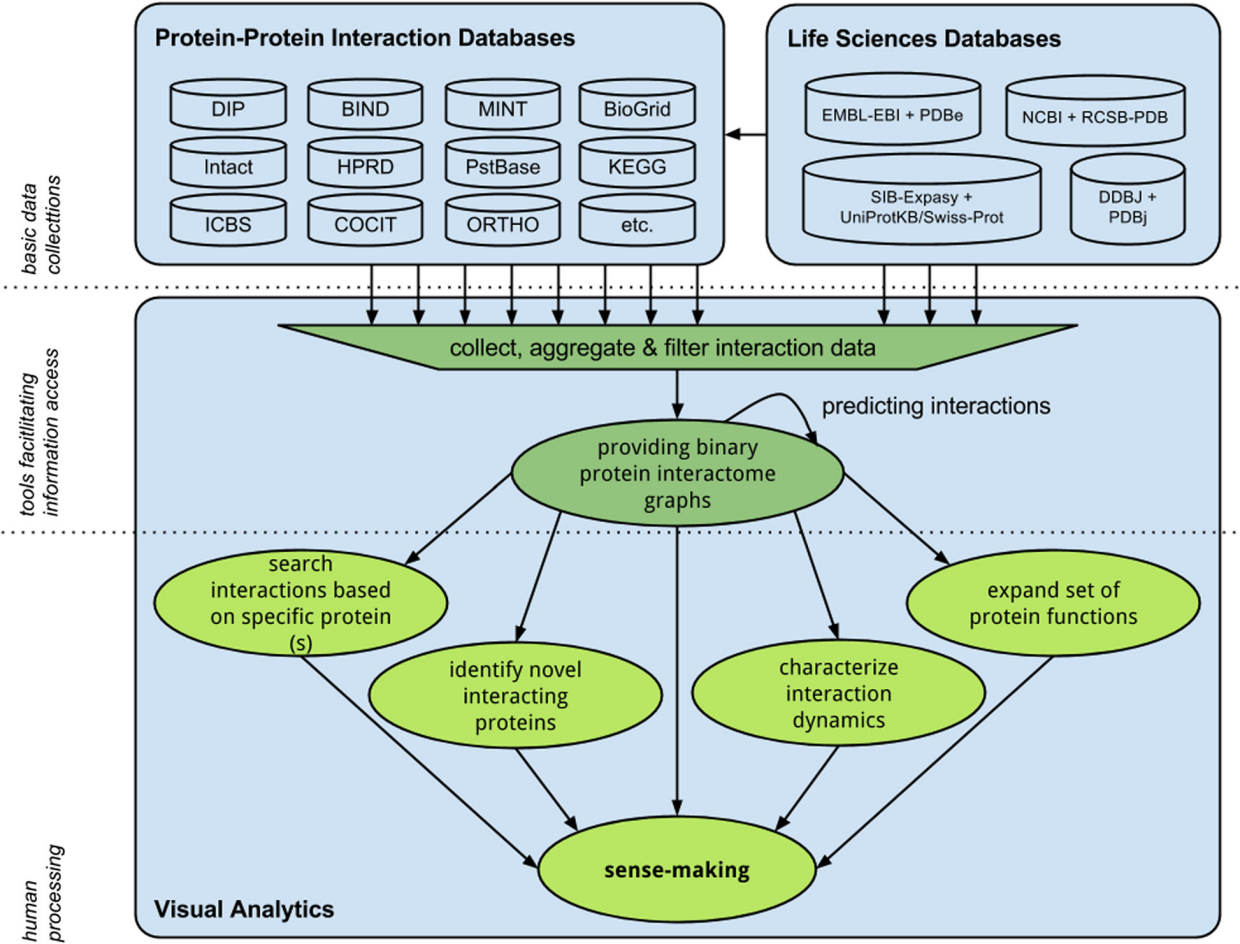
Process of visual PPI analysis

Figure 1 summarizes the visual analysis process. Current available biological databases contain huge quantities of different proteomic data that are used by tools to support the analysis process [3, 28]. Droit et al. [29] present an overview of different experimental and Bioinformatics methods to elucidate PPIs. Ben-Hur et al. [30] present computational approaches for prediction of PPIs to help experimentalists in the search for novel interactions. Mosca et al. [31] describe necessary steps towards a complete map of all human PPIs and list a set of currently available methods and resources for PPI analysis. There are several reviews and meta-databases of currently available interaction databases and tutorials on analyzing interaction data including [32–36], but none of these summaries depicts visualization features. Mora et al. [37] presents an analysis of some currently available software tools for PPI network visualization. However, the authors only focus on standalone software tools and do not include the analysis of web-based tools. Oveland et al. [38] review different proteomics software and depict exemplified visualization features for a wide range of proteomics data. The authors give a broad overview, but neither focus on PPI network analysis, nor provide a comprehensive overview of online available resources. There are also works that describe how to visualize protein interactions in three-dimensional space [39–42]. Regarding efficiency and effectiveness there are already some ongoing evaluations and efforts [4, 11, 15]. Several works also emphasize the importance of collaboration between computer science and biology [11]. For instance, PPI analysts would benefit from deepening studies not only in organizing and processing data, but also in text mining for protein function prediction as well as for enriching and combining different data and tools for extending association networks etc.

Computational systems biology assesses biological networks to analyze and visualize their complex connections computationally at a system-wide level [43]. In silico models have the purpose of replacing costly and time-consuming experiments with reconstruction and prediction by integration of the vast amount of biological information into multiscale computational modeling [44]. Modeling cellular networks in the context of physiological processes as well as diseases, including proteins as their major effectors, remains an exciting, open-ended domain [45]. Filling the gaps of missing data input by addition of literature-curated functional protein annotations poses a major task. Text-mining tools should help to analyze the overwhelming amount of literature [46]. Still, in regard to reliability and universality, tools require continuous improvements, for instance recognition of variable nomenclature and the implementation of ortholog-based annotations from conserved protein interaction graphs [47]. Biological management systems aim to provide user-friendly work-flows, shared to scientists, with integrated real-time visualization [5, 48].

To our knowledge there is no up-to-date comparative study of current tools that facilitate the interactive visual analysis of protein systems.

## Methods

We compare web-based resources for PPI analysis. 4 analysts take part in the evaluation. The interdisciplinary team consists of 3 domain experts from Computer Science and 1 from Biochemistry. 2 of the analysts are mentioned in the Acknowledgments. The other domain experts are the first 2 authors of this manuscript. We test the Bioinformatics resources by examining search user interfaces as well as visualization abilities. A checklist is completed during the test that includes qualitative meta-data and notes on usage. Additionally, several quantitative parameters are evaluated such as the number of links to different PPI sources, the total amount of PPIs, the number of search results for the specific query and other data if available.

We conduct a search for the “G Protein-Coupled Receptor Associated Sorting Protein 1” (GPRASP1), also known as “gasp1” with its UniProt ID “Q5JY77”. The example protein is chosen as input determinant due to its known involvement in G-protein coupled receptor (GPCR) signaling which constitutes a major cellular signal transduction cascade [49]. The cytosolic protein GPRASP1 is a validated tumor marker and, therefore, associated with cancer.[50]. Thus, we review the availability of information on disease associations. Additionally, we test for a set of proteins including GPRASP1 plus some of its putative interaction partners, namely cannabinoid 1 receptor CNR1 (P21554), calcitonin receptor CALCR (P30988), dopamine D2 receptor D2DR (P14416), bradykinin 1 receptor BDKRB1 (P46663) [49]. Results on the PPI searches regarding a single and multi-protein input are listed in Table 2.

We examine the presentation of results as well as visualization and interaction features. Quantitative and qualitative characteristics as well as notes are collected within spreadsheets. The results are summarized in a comprehensive comparison table (see link http://tinyurl.com/PPI-DB-Comparison-2015).

### Comparison Criteria

**Fig. 2:**
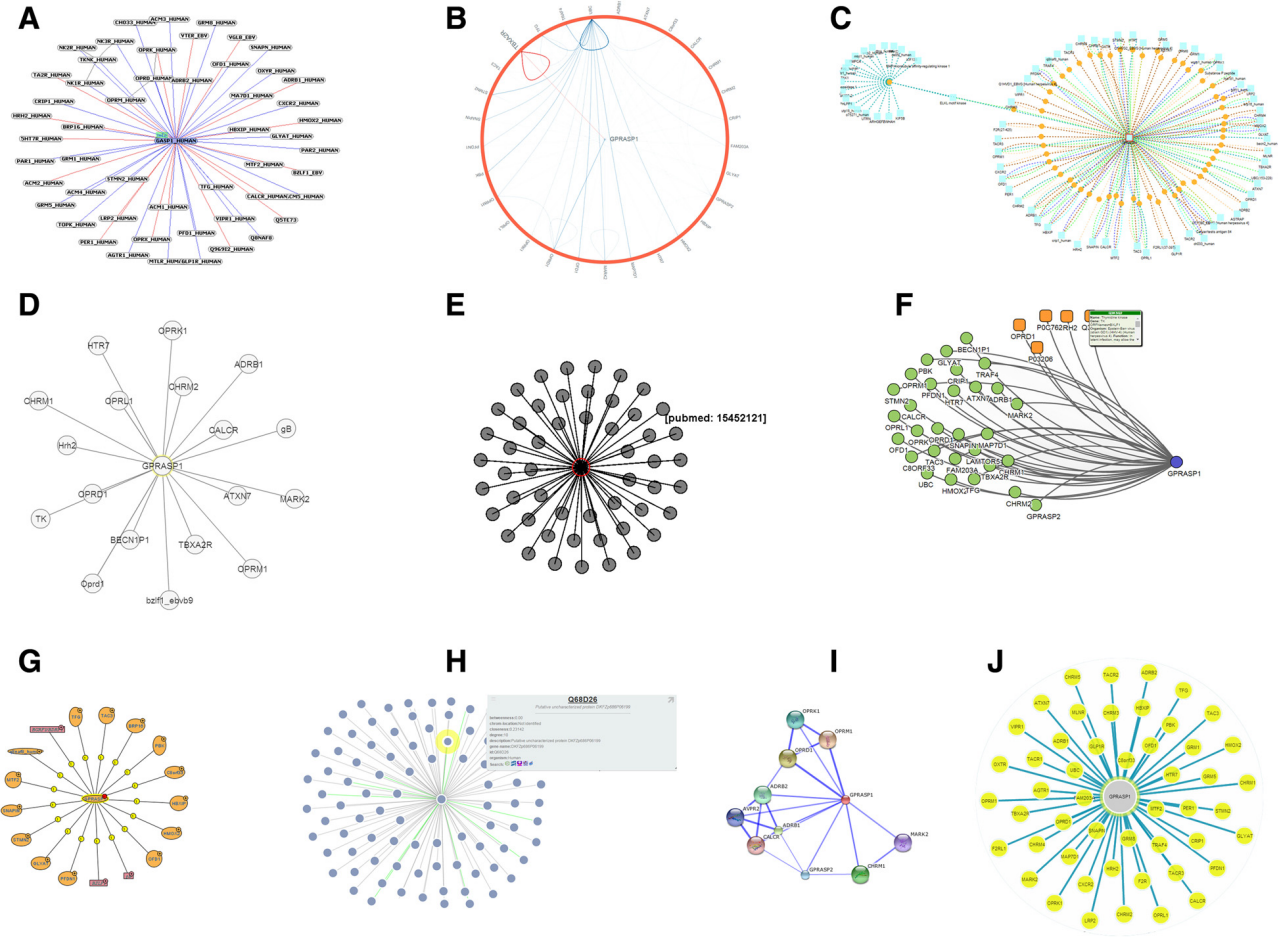
Graphical Comparison of all tools showing interactions networks for Q5Yj77: [A] APID, [B] Biogrid, [C] CPDB, [D] IntAct, [E] I2D, [F] Mentha, [G] MINT, [H] PINV, [I] String, [J] UniHI

Evaluations of visualization tools have to be prepared carefully. It is essential to choose an appropriate baseline for comparison and metrics by evaluating efficiency, effectiveness, visualization quality and insights. There are quantifiable factors such as speed (e.g. task performance), accuracy, latency, number of results, or insights. Additionally, there are standards for measuring qualitative factors that are currently used for the evaluation of research in clinical data visualization [51–54]. Some of these criteria are taken into account and are summarized for comparison. The review focuses on the following 5 criteria:

- **Support of Multi-Platform:** Nowadays research is conducted on miscellaneous devices, several operating systems and various browsers. Therefore, it is necessary to assess the requirements of a particular tool. Javascript and SVG are generally slower than Java applets or proprietary browser plugins such as Flash or Silverlight [55, 56]. None of the tested tools makes use of Silverlight at the frontend. Although Javascript often has shown performance problems in past, browser performance is rapidly evolving. Therefore, Javascript and SVG solutions can be used for graph rendering [20, 56–58].
  Next to a modern browser, end users often need to install plugins, including fFash. Java applets often need additional adjustments to the client’s security settings. Thus, Java applets but also Flash frontends (regardless whether based on Java or not) may pose a hurdle in making use of a visualization tool. Thus, Javascript and SVG visualization get the highest score for evaluating this criteria.
- **Service in General:** Determines the quality of the user interface (UI) in general. The UI determines the simplicity and efficiency of the search and its visualization characteristics.
- **Interoperatibility (Import, Export, Formats, Plugins):** Summarizes a tool’s network export options (e.g. textual, graphics, individual format), it’s interaction possibilities, manual import or similar options. This is particularly crucial when starting an analysis with one specific tool or one specific platform but continuing with another one.
- **Visualization Quality (Speed, Clarity, Usability):** Describes the visualization itself. Main focus lies on speed, clarity, and ease to use. This section also identifies items for possible improvement. In Fig. 2 all network views are compared to each other visually.
- **Visualization Features:** There are interactive visualization features that are crucial to exploration interfaces [12]. This section examines and lists available features like drag-and-drop, move background, area-selection a.o.
- **Data Coverage:** Represents the number of hits from the single and multi-protein search for PPIs as well as further information on associated diseases.

Each of the ten identified PPI web resources are tested against these criteria and the extent to which requirements are met for supporting the interactive visual analysis of PPI networks is evaluated. The evaluation summary comprises quantitative results such as the number of linked databases as well as the number of interactions found. Evaluation results also include last updates as important factor of comprehensiveness.

## Results

We specifically describe the most promising web resources. The visualization features of the selected resources are summarized in Table 1. Quantitative results are summarized in Table 2. We conclude with highlighting the top rated three resources that integrate the most promising interactive visualization features as well as integrate data comprehensively.

**Table 1:** Summary of identified PPI resources’ visualization control features

Tool ID/	Apid	BioGrid	CPDB	IntAct	I2D	Mentha	Mint	Pinv	String	UniHI
Control Feature										
Zoom	y	-	y	y	y	y	-	y	y	y
Select neighbors	-	-	y	y	y	y	-	-	-	-
Toggle labels	y	-	y	y	y	y	-	y	-	-
Fix/Unfix	-	-	-	-	y	y	y	y	y	-
Shrink/Grow	-	-	-	-	-	y	y	-	-	-
Toggle node shape	-	-	-	-	y	-	-	-	-	-
Select hubs	y	y	-	y	y	y	y	y	-	y
Select tree	-	y	-	-	-	y	-	-	-	-
Fit to screen	y	-	y	-	-	-	-	-	-	y
Clustering	-	-	y	-	-	-	-	y	y	y
Expand network	y	-	y	y	-	y	y	y	y	-

**Table 2:** Summary of the quantitative results concerning data integration

Tool ID/	Apid	BioGrid	CPDB	IntAct	I2D	Mentha	(Homo)Mint	Pinv	String	UniHI
Quantity aspect										
binary interactions	52	35	149,	22	53	35	17	95	201 (default 37)	50
of Q5JY77			(60 distinct)							
max. PPIs	322 579	543 666	368 654	473 426	1 539 758	480 517	330 377	n/a	332 235 675	374 833
							(Mint)			
human PPIs	83 670	173 728	221 328	154 338	318 717	157 932	241 458	2 942 636	942 636	n/a
							(HomoMint)			
predicted PPIs	44 040	n/a	n/a	n/a	635 488	n/a	6 782	n/a	n/a	n/a
experimental PPIs	278 539	n/a	n/a	n/a	922 617	n/a	323 595	n/a	n/a	n/a
group PPIs (Q5JY77,	91	n/a	4192	818	106	67	93	1894	470,	284
P21t4, P30988,									2 internal	
P14416, P46663)										
disease associations	n/a	n/a	n/a	0-2	n/a	0	n/a	n/a	13	0
links to DBs	29	12	32	27	29	6	6	1	23	15

The identified resources are: Agile Protein Interaction DataAnalyzer (APID) [59], BioGrid [10], ConsensusPathDB (CPDB) [60], IntAct - Molecular Interaction Database [21, 61], Interologous Interaction Database (I2D) [62], Mentha - The Interactome Browser [63], Molecular INTeraction database (MINT) [64], or more specific its’ separate annotation of human PPIs called HomoMINT [65], Protein Interaction Network Visualizer (PINV) [23], StringDB - Search Tool for the Retrieval of Interacting Genes/Proteins [66] and Unified Human Interactome (UniHI) [67].

### Agile Protein Interaction DataAnalyzer (APID)

**Support of Multi-Platform:** APID allows a protein’s interactions to be visualized as graph within a separate Java applet called ApinBrowser. Due to the usage of an embedded Java applet, the tool itself is multi-platform ready.

**Service in General:** APID allows queries of several input names. Results are presented in a concise way. Clicking on the number of interactions presents a more detailed overview of the PPIs including the number of experiments and information on sources of the various interactions. By clicking on the ’graph’ labeled button the Java applets are loaded into a separate window.

**Interoperatibility:** The tabular data can be exported. The graph itself can be stored as an image. Import possibilities are limited to searches throughout linked databases. The creators also provide a Cytoscape plugin for APID called APID2NET.

**Visualization Quality:** The visualization is dynamic and makes use of a simple force-based layout for graph drawing. It lacks anti-aliasing and other modern rendering techniques for visualization.

**Visualization Features:** APinBrowser provides options for zoom, filter and limiting details on demand. There are minor adjusting possibilities such as background color and edge thickness. Still, this resource lacks several features as visual clustering or highlighting certain nodes and edges.

**Data Coverage:** A single protein query quickly returns a mid-range number of interactions. Unfortunately, there is no direct option to include more than one protein name or ID into the search. However, after searching for one protein and visualizing the graph, it is possible to add additional proteins by using the “add” and “import” functionality within the applet. By further clicking on paint the additional proteins are included into the graph visualization. Associations to diseases are not available.

**Evaluation Summary:** The user interface of queries includes a concise tabular overview of results. Yet, anti-aliasing and options for adjusting nodes are missing. The web resource itself might be outdated due to the fact that last updates have been added in 2006.

### BioGrid

**Support of Multi-Platform:** This Bioinformatics resource can be opened in all current browsers. Therefore, installation of a specific plugin is not required.

**Service in General:** Biogrid provides a simple search option offering a quick glance on results in addition to filter and sorting features. The presentation of the results shows basic information.

**Interoperatibility:** The visualized graph can not be be exported. It can be downloaded as a simple textual list only. Additional download options can be found outside of the visualization view. However, a specific graph format for Cytoscape or similar tools is not included.

**Fig. 3:**
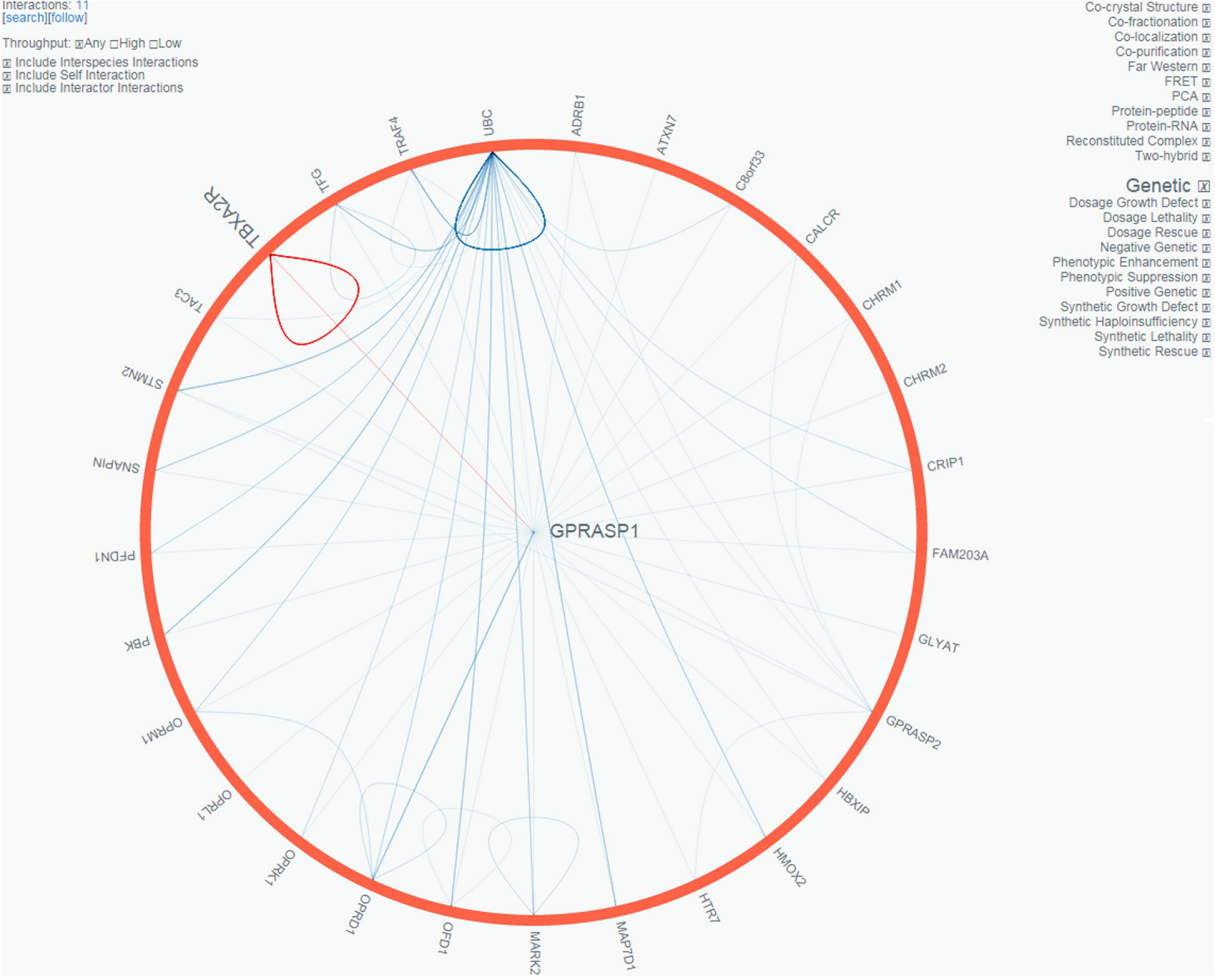
Screenshot of Biogrid’s graph view

**Visualization Quality:** The button for opening the graphical viewer is placed non-intuitively. The graph view loads quickly and does not require any plugin by making use of a modern circular layout that can be seen in Fig. 3. The radial view is not as intuitive as traditional graph presentations and the small labels are hard to read. Still, additional information is found quickly during the exploration process. There are no interactive features connected to the graph’s edges. By selecting a node, edges connected to this node are highlighted. During this process, the font size of the interacting nodes increases, that results in overlapping neighbors, rendering the text hardly readable. In terms of usability, the graph visualization provides features for basic analysis. Settings to adjust color and shape are missing.

**Visualization Features:** The visualization is static. The use of filtering options or other features forces the page to reload, which requires some computational time. Only exceptions are some hover effects. Rearrangement can be accomplished by clicking on a node. There are some features as highlighting, searching, filtering by the use of check-boxes and a field for input of text. Details are shown on mouse-over, also indicating the connected partners. Additional mouse-over details are options to search/follow interactions and download interaction data as text file. However, the visualization lacks zooming and scaling options.

**Data Coverage:** The single-protein query resulted in a low to mid-range number of interactions. Input options for a multi-protein search are not available, neither is information on disease associations.

**Evaluation Summary:** BioGrid supports visual analysis in a limited way.

### ConsensusPathDB (CPDB)

**Support of Multi-Platform:** Dynamic rendering of SVG visualization is possible in all modern browsers.

**Service in General:** CPDB offers an intuitive search combined with short computational loading times for the presentation of results. In addition, mapping criteria for filtering makes this resource a supportive PPI analysis tool.

**Interoperatibility:** CPDB is supported by only a small number of institutions unlike the other resources. Yet, it makes use of most important databases and offers features such as manual upload.

**Visualization Quality:** The network’s SVG based visualization is not as fancy as modern Flash based frontend presentations. Nevertheless, it already integrates anti-aliasing and interactiveness. CPDB provides many possibilities and includes many information sources. The graphs are largely and densely packed due to automatic stretching. The thickness of nodes does not correlate to the amount of visualized nodes. Their scale correlates with the zoom level, thus, the visualization becomes hard to read at a high zoom-level. The utilization of different colors and shapes facilitates a distinction between specific interaction- and node-types.

**Visualization Features:** Filter functions are not integrated into the visualization but have to be defined before mapping of interactions. The resource provides several criteria for mapping such as choosing particular databases to be integrated into the results. The dataset is visualized comprehensively. Additional information on nodes are shown by hovering and clicking on them. The network view makes use of zoom and repositioning options as well as color and shape differences of nodes and edges for highlighting certain attributes. The characters of shape and color are described in a concise and informative way within a legend. Edges can be merged and demerged. Network statistics can be retrieved and there is also a search option within the graph.

**Data Coverage:** CPDB shows the highest number of possible hits for both the single and multi-protein search. Information on associated diseases are not implemented.

**Fig. 4:**
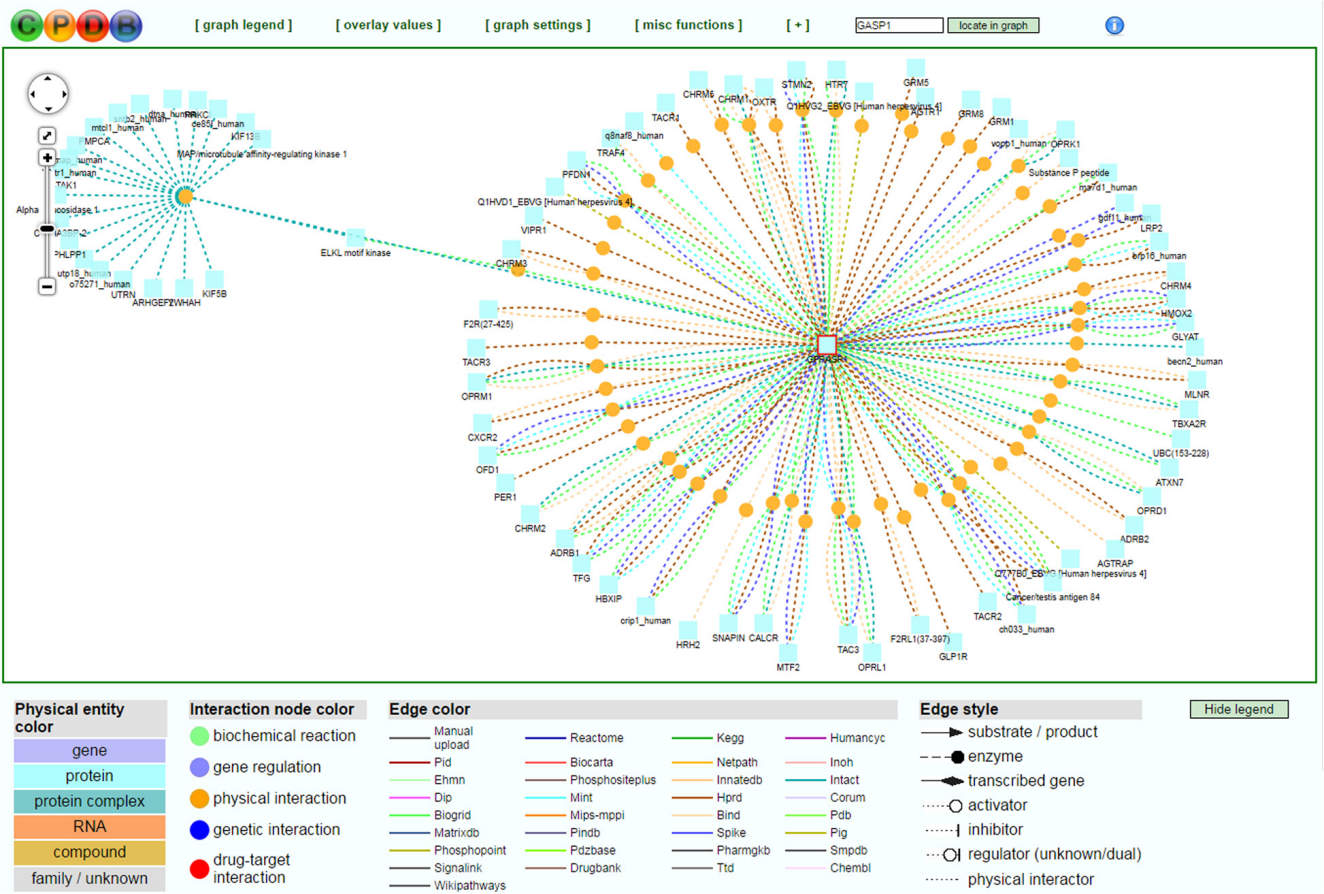
Screenshot of CPDB’s UI of interaction mapping and visualization

**Evaluation Summary:** CPDB holds the key benefit for supporting exploration by making use of PPI data obtained from literature curation, computational text-mining, orthology-based prediction as well as manual upload. Figure 4 presents a CPDB graph including interaction data, integrated in a merged manner. The developers try to avoid redundancies, still, the network visualization shows much more protein interactions compared to the other tools examined. On the one hand, CPDB’s graph presentation encourages exploration. On the other hand, there are difficulties of getting an overview.

### IntAct - Molecular Interaction Database

**Support of Multi-Platform:** The graph visualization is implemented via Flash. Flash has multi-platform support and is usable in all modern web browsers with installed Flash plugin.

**Service in General:** The search function is simple and intuitive. No preselection of attributes is necessary. Search results are presented as set of several subcategories.

**Interoperatibility:** PPI data within search results can be exported as tabular text. Additionally, the user can export export a network to the format of Cytoscape for further analysis and manipulation in the standalone tool.

**Visualization Quality:** The layout can be changed between force directed, radial and circular views. IntAct offers additional features as merging/splitting groups of nodes and zooming with modern anti-aliasing. However, IntAct lacks options for adjusting color and shape. There is a clear need for visual clustering, since every node looks the same. Titles of nodes are too large and occupy more area than the nodes themselves. Nodes overlap edges even in small graphs.

**Visualization Features:** There are several features as simple zoom and repositioning. Limited details are shown on demand by clicking on a node. The graph layout can be interactively adjusted. The user can switch between the list and the graph tab. Edges can be merged and demerged. Specific interactions can be filtered. Yet, there is no integration of detailed variations and highlighting specific variables.

**Data Coverage:** The single-protein query returns the low number of 22 possible PPIs, in case of protein ID as input, or 23 possible interactions in case of name abbreviation. IntAct presents one of the highest number in PPIs for the protein-group query. The feature of connecting to further EMBL-EBI resources reports associations of diseases in case of abbreviated name query.

**Evaluation Summary:** IntAct is supported by EBI and updated regularly. The integrated Flash based graph provides different export options including a translation to Cytoscape. However, the integrated visualization lacks important features such as filtering, adjustment of color and shape attributes.

### Interologous Interaction Database (I2D)

**Support of Multi-Platform:** I2D’s graph viewer needs Java installed and activated.

**Service in General:** The search option does not provide any auto-suggest and correction suggestions. The user has to search precisely. Other resources include such features. The table of results is very limited in information content, which only links to other meta-information on different platforms. No filter or sorting options are provided. It would be helpful to know the type of interaction at first sight.

**Interoperatibility:** There is only one possibility of inter-operating, as the graph can be exported as tabular text.

**Visualization Quality:** Due to the usage of an old fashioned Java applet the visualization lacks anti-aliasing and visualization quality. Nodes are covered by edges also in graphs with low numbers of nodes and edges. Rescaling options are missing.

**Visualization Features:** There are many hidden features that require parallel or cumulative actions with multiple input devices. A legend on key usage can be found on the right side within the network view. The legend is large and one example to the non-intuitive visualization approach.

**Data Coverage:** I2D presents a mid-range number of possible interactions for the single and multi-protein search. An option for disease association was not available.

**Evaluation Summary:** This resource links to many databases and therefore steadily expands its comprehensiveness. Still, the tool itself does not facilitate the process of visual analysis due to the outdated visualization integration.

### Mentha

**Support of Multi-Platform:** Mentha’s so called ‘interactome browser’ is implemented by Java. A newer but also limited SVG version is additionally provided as an alternative to Java.

**Service in General:** This Bioinformatics resource offers an intuitive search field but a less intuitive presentation of the results. The ‘browse’ button starts the network view. The ’list’ button itemizes interaction results and meta-information.

**Interoperatibility:** The new version does not provide export or import. The Java version supports export as textual tabular data and png graphics.

**Visualization Quality:** The SVG version is intuitive but still limited in optional features. Promising updates are already planned.

**Visualization Features:** The dynamic network viewer features zoom, filter details on demand and provides a flexible layout. Moreover, the Java version offers possibilities for coloring and highlighting.

**Data Coverage:** The interactome browser presents a low to mid-range number of possible interactions in case of the single-protein search and the lowest count in PPIs using the multi-protein input. Results can be easily filtered by confidence for a fast overview. The list is supplemented with meta-information from e.g. KEGG database and could offer associations to diseases but without any results from the particular evaluated search.

**Evaluation Summary:** There are several differences between the old and new visualization that are being integrated into Mentha. One comes with better compliance to the browser, the other one offers a higher degree of interaction possibilities. If being combined and steadily updated, the two visualization possibilities would definitely support the sense-making process. Future updates will include further enhancements to the new visualization.

### Molecular INTeraction database (MINT) / HomoMINT

**Support of Multi-Platform:** (Homo)Mint requires a browser with Java installed.

**Service in General:** The search UI provides a concise overview of results as well as includes an overview of the various databases used.

**Interoperatibility:** No import and export functions are integrated.

**Visualization Quality:** The resource is based on an old Java version does not integrate state of the art rendering techniques such as anti-aliasing. Most important interaction features are offered and performance is sufficient. A graphical legend is missing for a quick glance at means of color or shape.

**Visualization Features** Interaction possibilities include zoom, filter and details on demand. The user can change the size of nodes in order to improve speed and clarity. An adjustable threshold is available for filtering the output and number of displayed nodes. Drag and drop is possible (as in most other Java applets, too). Some features require a long computing time. One example is the option ’connect’ on a newly selected node for adding edges to it’s neighbors. Others are the MITAB and PSI functions. In this case, there are no notifications to the user. According to Nielsen’s response times, feedback should be provided after one second.

**Data Coverage:** Mint shows the lowest number of interactions for the single protein. Only 3 out of 5 proteins from the group input are detected and result into 93 PPIs after connecting the single graphs to each one of them. Information on associated diseases are available showing 3 interacting proteins out of 93 to be involved in pathological processes.

**Evaluation Summary:** Both quantitative (number of databases linked or number of interactions found) and qualitative results (old-fashioned visualization without anti-aliasing) underline the limitations of the Bioinformatics resource MINT. Since it is produced and provided by Uniroma, it is recommended to switch to the newer PPI tool supported by Uniroma: Mentha, which offers new visualization features, not limited to Java anymore.

### Protein Interaction Network Visualizer (PINV)

**Support of Multi-Platform:** The graph visualization runs in current browsers having Javascript installed and activated.

**Service in General:** The user interface for a query is intuitive. The idea of using the BioJS and D3 framework to create an HTML5 application, as it has been applied to this tool, offers interesting possibilities for supporting visual analysis online. However, performance limitations for large and dense graphs are still an issue when using the tool more intensely. Feedback often is missing at the right point and interaction possibilities could be smoother.

**Interoperability:** There are several possibilities to exporting the graph, both graphically and as textual tables.

**Visualization Quality:** Due to the increasing prospects of JS, the graph is rendered dynamically as SVG using anti-aliasing. This mode allows the user to interact with nodes and edges including smooth transitions. The default graph layout is a standard force-based view. In addition, PINV offers a circular layout, a heatmap as well as a simple table view.

**Fig. 5:**
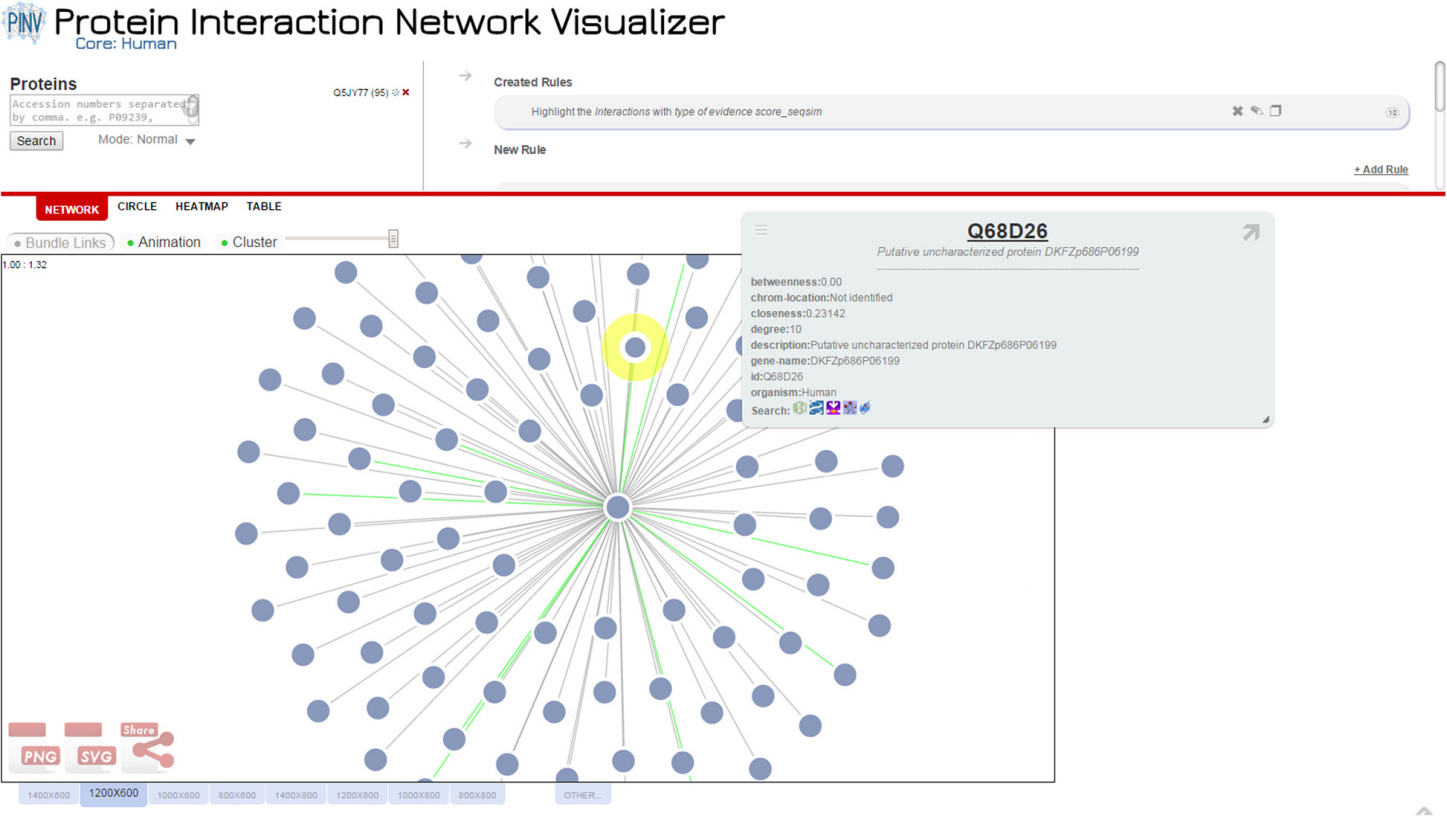
Screenshot of PINV UI showing search results for Q5Yj77

**Visualization Features:** The tool features several interaction possibilities, foremost zoom, filter and some details on demand. Next to the zoom option there are several possible manipulations to the visualization by defining rules for filtering, highlighting, coloring and options for uploading expression data. The screenshot in Fig. 5 illustrates that exploration is based on the process of defining rules.

**Data Coverage:** A suitable data-set has to be chosen from a list of online available sources before conducting protein search. By choosing the ’human’ data-set the single-protein input results into a higher count of 95 PPIs. One of the highest counts of 1894 PPIs follow from the multi-protein input. Further information on disease associations are not available.

**Evaluation Summary:** The visual analysis tool provides features for exploration and sensemaking in a modern fashion. Wizard-like usage and adding rules for manipulation can be recommended for other tools. Performance issues as well as not caught JS errors hinder the task of visual analysis of PPIs.

### StringDB - Search Tool for the Retrieval of Interacting Genes/Proteins

**Support of Multi-Platform:** StringDB’s interactive network viewer requires a modern browser including the Flash plugin.

**Service in General:** The query option is simple and includes data from several databases including multiple organisms.

**Interoperability:** The graph can be exported as several file formats, both as graphic and as text.

**Fig. 6:**
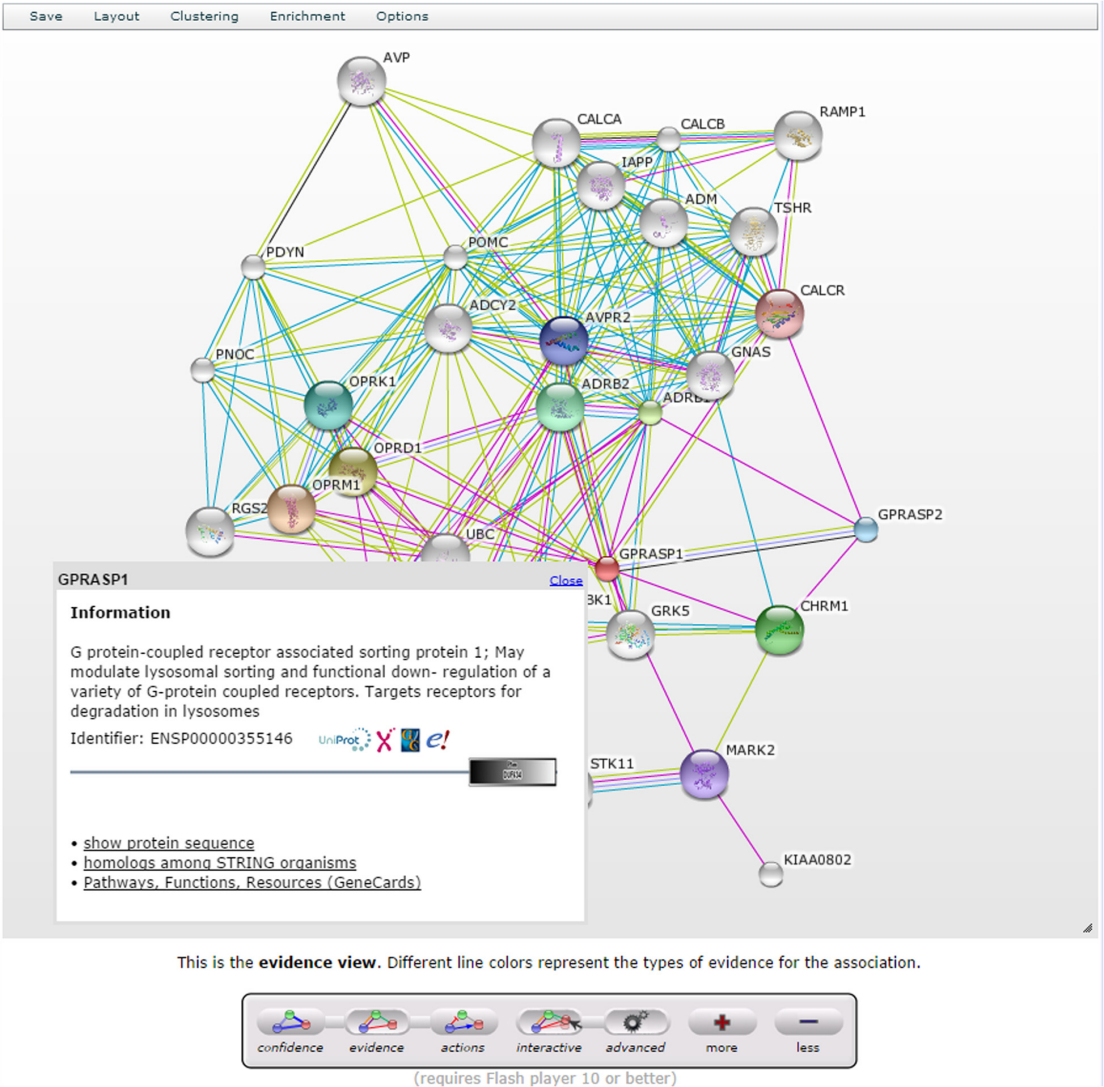
Screenshot of STRING UI showing the evidence view

**Visualization Quality:** Graphs are rendered dynamically as PNG ore implemented as interactive Flash visualization that offers numerous interaction possibilities. In addition to the network view, there are options for simple visualizations such as the occurrence view. Figure 6 illustrates some of StringDB’s UI capabilities. Further information as well as structural data are included if available. Details are displayed within the context menus upon clicking on individual nodes.

**Visualization Features:** The resource provides a variation of four different designs, namely confidence, evidence, actions and interactive view. The view can be changed from a simple default to an advanced mode. The interactive view allows the user to adapt the layout. The UI provides many different filter and control features next to simple zoom and scaling functionality. StringDB offers visualization options, such as node/label hide/show, and functional options of clustering or enrichment. The nodes and edges are colored. Node colors represent direct associations but are not adjustable. Line Colors are mapped to types of evidence. Line thickness represents confidence. These presentation presets are not customizable. The view does not allow zoom and is not adjustable in an arbitrary fashion. It provides options to grow and shrink the rendered image.

**Data Coverage:** The single protein query returns a mid-range number of hits, as does the multi-protein query. The default limit of reported interactions is set to 10 and has to be increased accordingly. Possible interactions are easily filtered by confidence. StringDB provides the option to get further information on disease associations. 13 associations are found within the 37 interacting proteins.

**Evaluation Summary:** StringDB combines comprehensiveness with state-of-the-art visualization features. It supports PPI visualization and analysis.

### Unified Human Interactome (UniHI)

**Support of Multi-Platform:** The graph visualization runs with Adobe Flash.

**Service in General:** The Java-based implementation needs to be improved regarding loading perfomance. The search UI is intuitive and easy to use. Still, tabs cannot be changed easily due to the UI’s implementation without hovering effects. UniHI links to several databases as common to most PPI resources. The graph visualization is rendered within the network tab.

**Interoperatibility:** Export options include text files, png and pdf.

**Visualization Quality:** The network visualization makes use of the common Cytoscape Web. This tool provides a modern but also simple Flash interface as frontend. The visualization encloses basic layout and filtering features that are capable of smoothly rendering large graphs. Unfortunately, the graph does not include any visual details. The visualization is rendered within a separate window. Selected or highlighted nodes are indicated by a lighter circle around the node. A separate menu at the right side of the resource includes filter and analysis features. Textual information is hard to read due to its’ small font-size. UniHI makes use of basic clustering or enrichment functions. Types of connections are colored differently within the visualization (red and blue). However, version 7.1 lacks functional layout palettes.

**Visualization Features:** The resource includes common control features such as zoom, repositioning and scaling to fit the page. It is possible to filter interactions (e.g. regarding source of interactions or amount of evidence). Details are provided in separate windows by clicking on a node. Analysis options are also provided. There are ‘Help’ links and a reset button for reconstructing the original graph setup.

**Data Coverage:** The single protein query yields a mid-range count in PPIs as does the multi-protein search. Information on target proteins are received from the KEGG database. In case of our query no implication on pathological associations could be detected.

**Evaluation Summary:** The old Java applet frontend has been upgraded to making use of Cytoscape’s Flash version. Yet, the resource does not meet the needs for exploration. Most of all, UniHI lacks performance and often throws irreproducible server errors that force the user to restart the query. Thus, UniHI cannot be recommended to support exploration as a step towards sensemaking.

## Discussion

We conducted an extensive web research and scanned through a list of more than 300 tools for PPI analysis. 53 are available online and suite the basic needs of protein system analysis within the human interactome. Only a small subset of the examined online tools (10 out of 53) offers integrated visualization. Interactive visualization features are summarized in Table 1. Quantitative metrics are summarized in Table 2.

At first glance, the primary goal of a search within web resources is to receive the largest amount of data. We quantified data retrieval by the number of possible interactions with a specific input variable. Therefore, web resources have to integrate data from several databases, and they have to be updated regularly. Ideally, data is obtained from several sources at once including literature curation, computational text-mining and prediction methods. A great amount of data does not equal a great deal of information. The search field and input options have to be easy to use. The user will stop his/her search at the initial stage if query options are not properly presented in the resource. Moreover, the presentation of data is crucial for its interpretation.

An ideal software tool for PPI analysis would possess the following features: At default results should be available as concise overview. Detailed information should become apparent on demand. Options for filtering and adjusting the confidence level are essential for a successful data translation. Graph visualization should be scalable and include features for manipulation. Nodes and edges exemplary should be adjustable in color, shape, size and position. Resources should offer various options to graph export and import. Results should be both complemented and downloadable as tabular text, graphics and also in other standardized file formats used by standalone tools. Above all, Bioinformatics web resources have to provide a modern interface. They have to comply with multi-platform standard browsers avoiding performance issues, outdated proprietary software, annoying software update requests or server errors.

In summary, the ideal web-based Bioinformatics resource features comprehensiveness, an intuitive user interface, as well as a modern visualization.

Each of the evaluated software has its respective strengths and weaknesses:

APID provides intriguing entry points such as a concise overview and a Cytoscape plugin. On the other hand, it lacks state-of-the-art rendering and modern visualization features like visual clustering.

Biogrid would benefit from improvements regarding readability and interactive features. Visualization would be ameliorated by making use of color and shape variations to visualize specific attributes. None of the test users found the option for opening the graphical viewer in Biogrid at first sight. This fact indicates the need for usability improvements.

CPDB presents a comprehensive dataset, while its visualization’s overview could be improved.

IntAct features an option for changing the network layout. However, it is only suitable to represent simple networks due to the lack of tagging and additional information.

I2D lacks state-of-the-art visualization quality and an intuitive and effective user interface. I2D’s user interface hinders exploration and sense-making.

(Homo)Mint provides interesting interactive visualization features like an adjustable threshold and drag and drop. Unfortunately, a graphical legend on feature description is missing. Some features require long computation times, and visualization quality is not state-of-the-art.

The idea of using JS frameworks such as BioJS and D3 in PINV is promising. However, PINV does not fully comply with the task of visual analysis of PPIs due to occurring performance issues as well as not caught JS errors.

StringDB’s presentation presets are not customizable yet. However, StringDB is our first choice of Bioinformatics resources due to its comprehensiveness, the use of confidence scores and state-of-the-art visualization features.

UniHI comes with two versions, a network view based on Java and another one running with Adobe Flash. The Java-based implementation needs to be improved regarding loading performance. Performance limitations are more likely to arise due to issues on server- and not client-side.

Force-directed layout is the main algorithm used in this kind of visualization tools. 2D graphs are the preferred solution for integrated visual analysis of PPI online. None of the tested tools features 3D views.

Only a few resources reasonably support exploration and sense-making. All identified web resources differ from standard graph visualization tools, mostly standalone software. Resources dedicated to PPI analysis also vary from graph analysis applications in other domains like link, social network or market analysis. Differences are observed in visualization quality and interaction possibilities. Therefore, export/import options are commonly implemented.

While conducting the evaluation of several online network visualization tools for PPI analysis we identified the following prominent challenges:

## Challenges

- **Challenge 1:** Current tools vary strongly in terms of comprehensiveness. Thus, it is still a crucial issue to link to all PPI databases available, finding suitable update mechanisms and providing a good overview in the distinct presentation of PPI networks.
- **Challenge 2:** Another only little-touched issue is dealing with confidence levels. Only a few tools provide the possibility to manipulate the graph drawing by adjusting the confidence of the various interactions as well as computing common metrics for graph network analysis. This is not only due to incompleteness of the underlying data used, but also because interactive features for visualization manipulation have long not been point of interest in the tool’s development.
- **Challenge 3:** A more general but also clear challenge deals with maturing visualization integration within the Biochemistry domain. There is a clear need to foster usage of modern visualization features such as easily changing layout settings, deleting nodes or adding group annotations, integrating richer possibilities for interactive visual clustering and extending layout palettes. The evaluation also highlights the need to also integrate, next to force-based algorithms, multi-level algorithms to overcome issues of assessing certain differences in networks and providing possibilities for presenting large graphs as both visually appealing and readable.

## Conclusions

The top three rated resources are String, IntAct and CPDB. They integrate graph visualization and can be successfully applied to interactive visual analysis of PPI. We also identified significant differences both in the UI as well as in the amount of hits on PPIs. Web-based resources are best used as starting point in research. Detailed analysis is still more efficient, effective and satisfying by making use of standalone graph visualization tools. This fact clearly reveals the necessity of further enhancing visualization integration in analysis tools in the domain of Biochemistry.

Closing, we encourage greater collaboration amongst the two scientific research fields of Systems Biology and Computer Science regarding visualization techniques.
